# Association of comorbidities and drug intake with generalized chronic pruritus: A multicenter case-control study in Switzerland and Italy

**DOI:** 10.1016/j.jdin.2025.08.007

**Published:** 2025-09-04

**Authors:** Michael Benzaquen, Simone Cazzaniga, Franco Rongioletti, Elena Pezzolo, Giorgia Di Marco, Luigi Naldi, Luca Borradori

**Affiliations:** a Department of Dermatology, Inselspital, Bern University Hospital, University of Bern, Bern, Switzerland; b Centro Studi GISED, Bergamo, Italy; c School of Medicine, Vita-Salute San Raffaele University, Milan, Italy; d Dermatology Clinic, IRCCS San Raffaele Scientific Institute, Milan, Italy; e Department of Dermatology, AULSS8 Ospedale San Bortolo, Vicenza, Italy

**Keywords:** association, chronic pruritus, comorbidities, medications, risk factor, systemic diseases

*To the Editor:* Chronic pruritus (CP), defined as itch lasting 6 weeks or longer, is a distressing symptom often associated with systemic diseases, drug intake, or psychosocial factors.^1^^,^^2^ Despite its prevalence and clinical burden, evidence on non-dermatologic triggers remains limited.^3^^,^^4^ We conducted a prospective multicenter case-control study to identify comorbidities and medication exposures associated with generalized CP while excluding patients with identifiable pruritic dermatoses.

From January 2022 to December 2024, adult patients (≥18 years) with generalized CP were enrolled across 3 dermatology centers in Switzerland and Italy. CP was defined as itching affecting at least 3 different body regions for 6 or more weeks. Patients with active dermatologic causes were excluded based on clinical and, when necessary, histopathologic evaluation. A small subset had a remote history of atopic dermatitis, but without current clinical activity. Chronic scratch lesions were considered *secondary to itch* and not exclusionary.

Each case was matched (1:2) by age (±5 years) and sex to control patients without current or past CP, presenting for non-pruritic dermatologic concerns. We collected demographic, clinical, and medication data (Table I). Medication exposure was defined as use of a drug for ≥6 weeks in the 12 months preceding itch onset. Multivariable conditional logistic regression was used to identify independent associations.

**Table I:** Sociodemographic characteristics of cases and controls

Sociodemographic characteristics	Controls		Cases		*P*∗
	*N* = 154	%	*N* = 77	%	
Sex					
Male	76	49.4%	38	49.4%	-
Female	78	50.6%	39	50.6%	
Age at the visit date (y)					
Mean, SD	65.2	16.1	65.8	16.8	.15
Age at the index date†(y)					
Mean, SD	65.2	16.1	62.6	18.1	.002
BMI (kg/m^2^)					
Mean, SD	25.2	3.7	24.9	4.4	.58
< 25.0	83	53.9%	45	58.4%	
25.0 - 29.9	56	36.4%	24	31.2%	
30.0+	15	9.7%	8	10.4%	
Occupational status					
Working	61	39.6%	29	37.7%	.95
Unemployed	11	7.1%	7	9.1%	
Retired	74	48.1%	37	48.1%	
Invalid/disabled	8	5.2%	4	5.2%	
Highest level of education					
Compulsory education	39	25.3%	29	37.7%	.04
High school	61	39.6%	32	41.6%	
University	54	35.1%	16	20.8%	
Smoking habits					
Smoker	33	21.4%	16	20.8%	.78
Ex-smoker	52	33.8%	23	29.9%	
Non-smoker	69	44.8%	38	49.4%	
No. cigarettes per day					
Mean, SD	18.2	11.3	16.6	12*.*2	.95
Alcohol consumption					
Regular drinker	20	13.0%	16	20.8%	.01
Occasional drinker	79	51.3%	29	37.7%	
Ex-drinker	19	12.3%	3	3.9%	
Non-drinker	36	23.4%	29	37.7%	
Substance abuse					
Hard drugs	1	0.6%	0	0.0%	.68
Soft drugs	6	3.9%	1	1.3%	.31
Other kind of soft drugs	0	0.0%	1	1.3%	.58

*BMI,* Body mass index; *SD*, standard deviation.

∗ Differences between cases and matched controls were assessed by univariate conditional logistic regression analysis.

† The index date was, for cases, the date of starting of itching and, for controls, the date of first onset of symptoms or lesion detection leading to the visit.

A total of 77 CP cases and 154 controls were included. Most patients with CP were categorized as having pruritus on non-diseased skin (40.3%) or CP with chronic scratch lesions (39%). In 54.5%, an underlying systemic cause was suspected; 37.7% remained idiopathic. Mean pruritus intensity was 7.9/10 (SD: 2.3), burning sensation (mean: 4.8, SD: 3.6), and stinging sensation were mild (mean: 3.1, SD: 3.4).

Significant comorbidities (Table II) more frequent in cases included diabetes mellitus (23.4% vs 12.3%), allergic conditions (20.8% vs 5.2%), digestive disorders (23.4% vs 9.1%), and chronic infections (11.7% vs 0.6%). Infections included non-dermatologic conditions such as chronic H. pylori gastritis, hepatitis C, and recurrent urinary tract infections—*not* tinea or scabies, which were excluded. History of cardiovascular disease was evaluated but not independently associated with CP after adjusting for medication use.

**Table II:** Lifetime comorbidities and medications used for at least 6 weeks in the last 12 months before the index date, among cases and controls

Comorbidities and medications	Controls		Cases		*P*∗
	*N*	%	*N*	%	
General comorbidities					
Thyroid	22	14.3%	14	18.2%	.09
Anaemia	1	0.6%	12	15.6%	.06
Iron deficiency anaemia	1	0.6%	8	10.4%	.12
Other anaemia	0	0.0%	6	7.8%	.18
Diabetes mellitus	19	12.3%	18	23.4%	.03
Type I diabetes	6	3.9%	5	6.5%	.38
Type II diabetes	13	8.4%	13	16.9%	.06
Arterial hypertension	46	29.9%	31	40.3%	.08
Hyperlipemia	35	22.7%	28	36.4%	.02
Allergic conditions†	8	5.2%	16	20.8%	.001
Renal	5	3.2%	7	9.1%	.07
Liver	9	5.8%	8	10.4%	.22
Neoplastic malignancies	22	14.3%	10	13.0%	.79
Paraproteinemia	1	0.6%	1	1.3%	.62
Rheumatic	15	9.7%	12	15.6%	.18
Neurological	7	4.5%	7	9.1%	.19
Cardiovascular	24	15.6%	20	26.0%	.06
Haematological‡	4	2.6%	1	1.3%	.54
Hypereosinophilia	0	0.0%	3	3.9%	.34
Digestive§	14	9.1%	18	23.4%	.006
Infections‖	1	0.6%	9	11.7%	.006
Others	18	11.7%	6	7.8%	.32
Skin comorbidities					
Atopic dermatitis	1	0.6%	4	5.2%	.06
Contact dermatitis	8	5.2%	5	6.5%	.66
Urticaria	0	0.0%	11	14.3%	.07
Psoriasis	2	1.3%	2	2.6%	.49
Acne	0	0.0%	1	1.3%	.58
Rosacea	1	0.6%	2	2.6%	.26
Others¶	9	5.8%	2	2.6%	.30
Medications					
Antidiabetics	19	12.3%	17	22.1%	.07
Anticoagulants	9	5.8%	8	10.4%	.18
Xa inhibitors	6	3.9%	4	5.2%	.64
Thrombin inhibitors	4	2.6%	4	5.2%	.33
Diuretics	7	4.5%	10	13.0%	.02
Neuroleptics	5	3.2%	5	6.5%	.25
NSAIDs	6	3.9%	7	9.1%	.11
Antiarrhythmics	7	4.5%	14	18.2%	.002
Antidepressants	2	1.3%	8	10.4%	.14
Antihypertensives	43	27.9%	29	37.7%	.09
Antilipemics	21	13.6%	18	23.4%	.06

∗ Differences between cases and matched controls were assessed by univariate conditional logistic regression analysis.

† Including allergic rhinitis, asthma, drug or food allergy.

‡ Including haematological malignancies.

§ Including benign neoplasm of colon, gastro-esophageal reflux disease, Barrett's esophagus, gastric ulcer, gastritis, diaphragmatic hernia, ulcerative colitis and rectosigmoiditis, diverticular disease of intestine, calculus of gallbladder, pancreatic disease, dysphagia.

‖ Including tuberculosis, herpesvirus infection, varicella, infectious peritonitis, encephalitis.

¶ Excluding skin cancer.

In fully adjusted models, CP was significantly associated with.

• *Diabetes mellitus* (OR: 9.93; 95% CI: 2.44-40.43)
• *Allergic conditions* (OR: 15.9; 95% CI: 2.65-95.28)
• *Digestive disorders* (OR: 4.26; 95% CI: 1.04-17.43)
• *Chronic infections* (OR: 16.24; 95% CI: 1.003-263.0)
• *Atopic dermatitis* (past history, no active lesions) (OR: 94.86; 95% CI: 2.42-3714)
• *Use of diuretics* (OR: 11.61; 95% CI: 1.35-99.59)
• *Use of antiarrhythmics* (OR: 5.75; 95% CI: 1.14-28.97)
• *Lower education levels* (compulsory: OR: 5.28; high school: OR: 3.37 vs university)

While many findings confirm established associations,^5^ our study identifies *novel links with digestive disease, chronic infections*, and *socioeconomic factors*. The modest sample size reflects strict inclusion criteria and prospective recruitment but limits power to detect weaker associations.

In summary, generalized CP is associated with systemic comorbidities, specific drug classes, and social determinants of health. Thorough evaluation of medical history, medication use, and psychosocial context is critical for effective diagnosis and management of CP.

## Conflicts of interest

None disclosed.
